# Removal of Heavy Metals from Wastewaters: A Challenge from Current Treatment Methods to Nanotechnology Applications

**DOI:** 10.3390/toxics8040101

**Published:** 2020-11-10

**Authors:** Ruxandra Vidu, Ecaterina Matei, Andra Mihaela Predescu, Badriyah Alhalaili, Cristian Pantilimon, Claudia Tarcea, Cristian Predescu

**Affiliations:** 1 Faculty of Materials Science and Engineering, University Politehnica of Bucharest,060042 Bucharest, Romania or rvidu@ucdavis.edu (R.V.); cristian.pantilimon@ecomet.pub.ro (C.P.); claudia.dragan@ecomet.pub.ro (C.T.); predescu@ecomet.pub.ro (C.P.); 2 Department of Electrical & Computer Engineering, University of California, Davis, CA 95616, USA; 3 Nanotechnology and Advanced Materials Program, Kuwait Institute for Scientific Research, Kuwait City 13109, Kuwait; bhalaili@kisr.edu.kw

**Keywords:** heavy metals, nanotechnology, toxics, wastewaters, nanomaterials, water treatments, electrochemistry, removal, detection, template synthesis, nanostructure array

## Abstract

Removing heavy metals from wastewaters is a challenging process that requires constant attention and monitoring, as heavy metals are major wastewater pollutants that are not biodegradable and thus accumulate in the ecosystem. In addition, the persistent nature, toxicity and accumulation of heavy metal ions in the human body have become the driving force for searching new and more efficient water treatment technologies to reduce the concentration of heavy metal in waters. Because the conventional techniques will not be able to keep up with the growing demand for lower heavy metals levels in drinking water and wastewaters, it is becoming increasingly challenging to implement technologically advanced alternative water treatments. Nanotechnology offers a number of advantages compared to other methods. Nanomaterials are more efficient in terms of cost and volume, and many process mechanisms are better and faster at nanoscale. Although nanomaterials have already proved themselves in water technology, there are specific challenges related to their stability, toxicity and recovery, which led to innovations to counteract them. Taking into account the multidisciplinary research of water treatment for the removal of heavy metals, the present review provides an updated report on the main technologies and materials used for the removal of heavy metals with an emphasis on nanoscale materials and processes involved in the heavy metals removal and detection.

## 1. Introduction

In the rapid global industrialization, there is a constant effort to improve the use-reuse cycle of water and to protect water resources through legislation, which is the driving force for research and innovation. In addition to numbers and statistics, there is the health factor that cannot be quantified when it comes to people. Water pollution with heavy metals is one of the most harmful pollution throughout the globe due to their non-degradable properties. Despite the national and international standards stipulated by the World Health Organization (WHO) and the United States Environmental Protection Agency (USEPA) that the drinking water should not exceed a maximum concentration of a few to a few tens of µg/L [1,2], heavy metals can accumulate in the ecosystem and enter the human body through food. Table 1 summarizes the acceptable limitations of various heavy metals according to the World Health Organization [2] and the United States Environmental Protection Agency (USEPA) [1]. That is why the need for efficient, fast, reliable and accurate methods to completely remove heavy metals from wastewaters including sewage irrigation, exhaust emissions, and mining became increasingly important for our safe drinking water.

**Figure 1:**
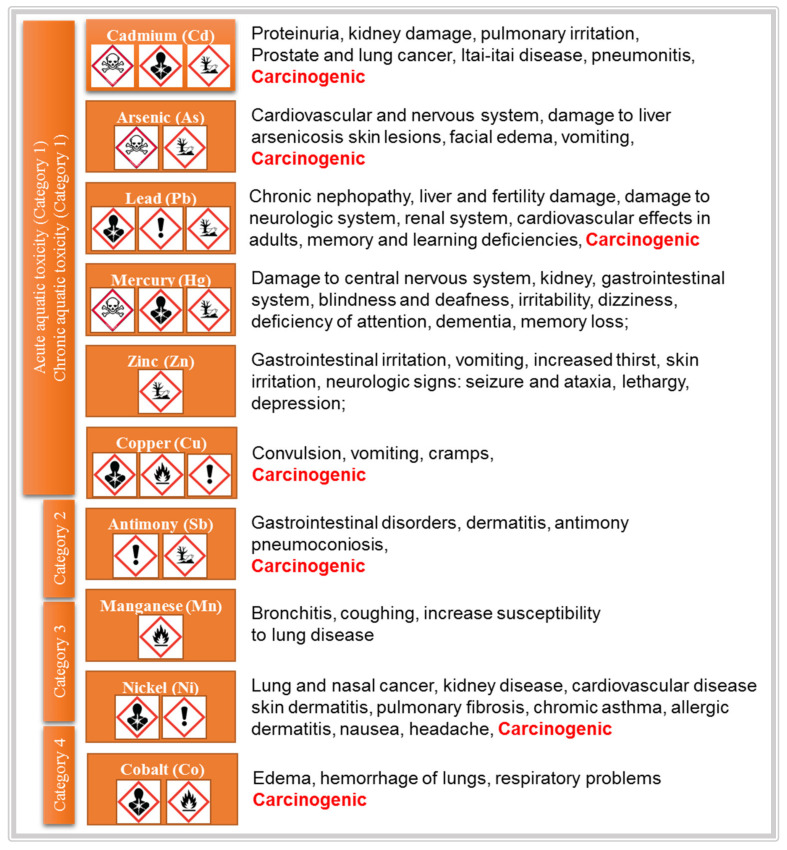
Illustration of the main heavy metal ions (HMIs) present in wastewaters, which are listed according to their toxicity, with pictograms and main health hazards for each element, according to the United Nations Guide to the Globally Harmonized System of Classification and Labeling of Chemicals (GHS) [3].

Because heavy metals do not degrade in nature, they contaminate natural resources. In waters, heavy metals such as cadmium, chromium, arsenic, lead and mercury are highly toxic for human health even at trace levels (Figure 1). In concentrations higher than a few µg/L, heavy metals affect the normal development and function of organs, poisoning the human body and damaging internal organs and tissues by various mechanisms such as enzymes denaturation, ions replacement and proteins inactivation.

Because wastewater effluents show different severities in terms of contamination with pollutants, they are treated differently depending on their specific use. Pathogens and heavy metals represent factors that are unwanted in the water discharge from treatment plants, but certain elements such as nitrates and phosphorus can be beneficial if the effluent is used for agriculture, or they can be detrimental if the water is dumped into rivers [4,5].

Therefore, the development of tools and technologies to quantify, analyze and monitor heavy metals has become as important as the development of new technologies for the removal of heavy metal ions (HMIs) from waters. Nanotechnology can bridge the instrument-based analysis and technologies for HMIs removal. Santhosh et al. [6] have extensively reviewed the performance of nanomaterials in water treatment methods that make use of the adsorption, photocatalytic and antibacterial activity of nanomaterials. Advanced oxidation processes are technologies for the treatment of wastewaters and effluents with refractory organic pollutants that show future promise [7] and that use semiconductor photocatalysts to remove a large number of toxic pollutants.

Electrochemistry is an interdisciplinary study of the charge transfer at interfaces, which has a contradictory duality in the case of water treatment, and more specifically of the removal of HMIs from wastewater, i.e., it is both a source of heavy metals and a remedy. Electrochemistry is a source of water pollution because there are industries that are based entirely on electrochemical processes such as electroplating industries that dispose their solutions containing Zn, Pb, Cu, Cd, Cr and cyanides into the waters and the battery industry that poisons the waters with Pb, Hg and Cd. Besides water pollution from industries, there is corrosion, which is also an electrochemical process that contaminates the water with heavy metals coming from the metal plumbing, usually Pb. Miniaturization of electronics and devices is the driving force in the development of integrated nanostructures [8,9].

**Figure 2:**
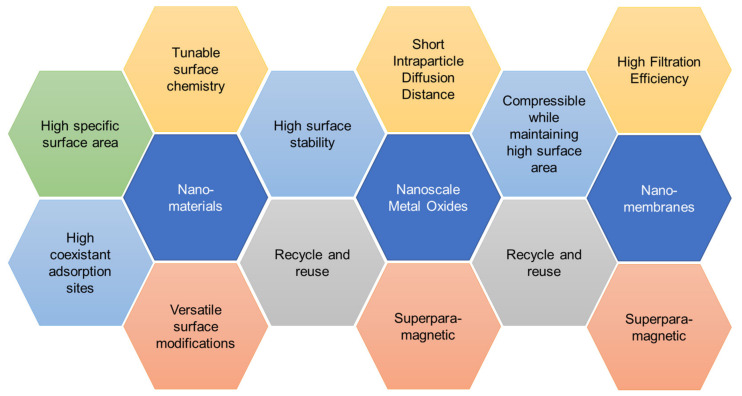
Nanomaterials and their surface properties that can be control in various applications.

Whether it is nanomaterials, nanoparticles or nanomembranes, they do share the same properties and characteristics. Figure 2 shows how the main surface properties of nanomaterials and their unique characteristics combine and work together to confer them higher efficiency than the same bulk material. In Figure 2, nano-based materials used in adsorption studies, nanoscale metal oxides used in photocatalysis and nanoporous membranes for filtration and desalination are shown in the middle row, while their tunable properties, process variable and intrinsic properties are represented as satellite hexagons around them. This figure illustrates the complex and sometimes unique combination of variables, which is critically important in understanding the relationship between nanomaterial, composition and properties for high performance in a given application.

Several reviews have been published on water remediation for HMIs removal. Babel et al. [10] presented a critical analysis of the application of chemical extraction, bioleaching, electroreclamation, and supercritical fluid extraction, in removing heavy metals from contaminated sludge. Hua et al. [11] have extensively reviewed nanostructured metal oxides and porous host supported nanoparticles including their synthesis, physicochemical properties, adsorption characteristics and mechanism, as well as their application in HMIs removal. In a more recent review, comprehensive information on different adsorbents that are used for heavy metal removal, as well as on the commercially available and natural bioadsorbents used for removal of Cr, Cd and Cu, are provided [12].

Because of the multidisciplinary aspect of the research on the removal of heavy metals, the present review provides an updated report on the nanoscale materials and processes involved in the heavy metals’ removal and detection. New developments in the field of water treatment as well as existing technologies and future potential of those technologies that could greatly benefit from implementation of recent nanotechnology developments are presented. There are great opportunities for nanotechnology to clearly impact this area of water treatment and bridge various techniques to improve large scale applicability nanoscience-based technologies for HMIs removal.

## 2. Chemical and Physical Methods

### 2.1. Chemical Precipitation

Chemical methods are extensively used in water treatment for retaining metals, inorganic compounds, oils, fats and different organic materials from wastewaters. Precipitation is a process of leading pollutants, which are dissolved or suspended in solution, to form a solid precipitate which can be later separated from the liquid. The ions and particles can be easily removed with a large precipitate. Coagulants such as polymers are used in order to gather the small suspended particles into larger precipitates. The polymer molecules can be charged or neutral (i.e., cationic, anionic or nonionic). Because of the interaction between ions and charged particles in liquid, the polymers behave either as bonds between particles or particles neutralizer in solution. Depending on the contaminants that have to be removed, specific precipitation methods can be used [13].

#### 2.1.1. Hydroxide Precipitation

In hydroxide precipitation, soluble heavy metal ions are converted into insoluble metal compounds by adding hydroxide. The aim of metals treatment by hydroxide precipitation is to form precipitates by adjusting the water pH. Theoretical and experimental studies were performed by Tunay et al. [14] to understand the mechanism of ligand-sharing effect of metals, which are added to wastewater to ensure effective removal of complexed heavy metals. This work indicated that high pH precipitation occurred when organic ligand can be effectively bound by a coagulant or pH adjustment agent, thus freeing the heavy metal to form hydroxide or carbonate solids [14]. After metal precipitation and solids forming, they can be very easily removed from waters. The main factors influencing the metal precipitation are the metal concentrations and water pH. Hydroxide precipitation is efficiently used in the case of small quantities of heavy metals in water (1–100 mg/L) and in acidic or neutral pH media.

#### 2.1.2. Sulfide Precipitation

A more complete heavy metals removal from wastewaters is provided by sulfides rather than hydroxides for the precipitation process. Usually, metal sulfides possess solubility several orders smaller in comparison with the corresponding hydroxides, making this technique superior to the hydroxide precipitation. Unlike hydroxide precipitation, sulfide precipitation is not sensitive to the presence of many chelating agents or complexes. Chromates and dichromates removal take place without the initial step of chromium reduction to Cr(III) [15]. Sulfide precipitation with Na_2_S was found to be highly effective in the removal of Cd, Zn and Cu from wastewaters by more than 99%, and As and Se removals by more than 98 and 92%, respectively [16]. The resulting sulfide metal sludges are better compressed and more stable than the hydroxide precipitation, demonstrating better dewatering and density characteristics.

#### 2.1.3. Other Techniques Using Chemical Precipitation

Other reagents used in wastewater treatment by chemical precipitation are as follows:

- Ferrous sulfate is usually used together with lime for water softening. The aggregates consist of calcium sulfate and ferric hydroxide. The condition for a successful removal process is that the wastewater must contain dissolved oxygen that is required by the chemical reaction to form the precipitate [17].
- Aluminum for the removal of phosphate and water softening. The reaction takes place with phosphate or various alkaline compounds (i.e., bicarbonate, hydroxide or carbonate) that form insoluble aluminum slats [17].
- Ferric chloride forming insoluble iron salts together with phosphates or alkaline compounds [17,18].
- Polymers, which can be anionic, cationic or nonionic. They can be used for neutralization or as links coagulants after they are added to wastewaters [19].

Chemical precipitation has been used in order to remove heavy metals from municipal and industrial waters. Even though the chemical precipitation is a well-established technique, today’s research concentrates on merging this method with different other treatment techniques such as reverse osmosis and photochemical oxidation in order to boost the efficiency of heavy metal removal from wastewaters [20]. Table 2 presents the advantages and disadvantages of the chemical precipitation function of the coagulant used in the process.

### 2.2. Ion Exchange

Ion exchange process represents an efficient tool in the environmental protection with waste waters application. Over the last years, new or improved classical materials were tested as ion exchangers [21]. A large class of pollutants as metal ions could be identified in liquid effluents from industrial to nuclear [22,23,24,25,26,27] such as Cd^2+^, Pb^2+^ and Hg^2+^, as well as radionuclides as ^137^Cs, ^89^Sr, ^235^U, ^59^Fe, ^57^Co, ^65^Zn and anionic species: CrO_4_^−2^ and AsO_4_^−3^ [23,25]. For all these metal ions, a variety of removal methods were tested from precipitation [26], as an inexpensive and simple technique, to biological techniques with high costs and the same disadvantage regarding high quantity of sludge generation, such as precipitation [24]. Moreover, Hoch and co-workers reported good results with photocatalysis technique, but they identified the risk of appearance of undesirable compounds such as Cr(III) from Cr(VI) that is necessary to be removed [22].

In this context, ion-exchange technique is recognized as adequate and cost-effective technique, with high possibility for regeneration of used exchangers. Usually, conventional materials such as organic polymers or inorganic zeolites, clay are used as exchanger materials [28,29,30]. The removal capacity through ion exchange depends on chemistry selectivity of species, for example, organic materials with sulfonate, amino, thiol or amide functional groups are applied for water treatment with cadmium and nickel removal [31,32].

With the same efficiency, polymeric with carboxylate groups are able to removed Cd, Ni, Cu and Pb as traces from waste waters [33,34,35,36,37]. Even if there are some good results within these conventional materials, a few disadvantages are important enough to be mentioned: low capacity of reuse, kinetics and chemical and thermal stability for organic resin reuse [38,39,40,41,42].

This is the reason for which appears as a necessity to identify and testing new materials with ion exchange capacity. Thus, in the last years, the tendency was to obtain and test excellent materials, which expose multiple functional groups, high surface area and chemical and thermal stability [43,44,45,46,47]. In this context, metal-organic ion exchange materials are found as a combination between conventional materials with great porosity and binding groups [21,48,49]. An example of this type of materials could be zeolite nanoparticles impregnated with polysulfone used to remove Pb and Cd from waste waters [21].

### 2.3. Adsorption

#### 2.3.1. Carbon-Based Adsorbents

Absorbents are generally characterized by heterogeneous and irregular surfaces, which promote the adsorption of HMIs. After the sorption of pollutants, the surface becomes smoother. When the impregnation with nanoparticles is used to modify the surface of adsorbents, macro-clusters are formed while the surface becomes more regular. Further surface treatment processes such as carbonization and calcination return the adsorbent surface to porous. Although other surface modifications such as chemical modification reduce the total surface area, they offer control on adjusting the functional groups in order to increase the adsorption capacity. Yang et al. [50] have recently reviewed the effects of various functional groups on HMIs adsorption onto different carbon materials, emphasizing the importance surface modification of carbon adsorbents in optimizing their physicochemical and sorptive properties in the development of new technologies for environmental remediation.

#### 2.3.2. Carbon Nanotube (CNT) Absorbents

Table 3 presents a summary of the maximum adsorption capacities for some heavy metal ions on multiwalled carbon nanotubes (MWCNTs), single-walled carbon nanotubes (SWCNTs), oxidized CNTs and activated charcoal (granular activated charcoal (GAC) and powder activated charcoal (PAC).

Table 3 shows that the removal of Ni^2+^ by oxidized SWCNTs, (MWCNTs) and granular activated carbon (GAC) [58]. These adsorbents were investigated under the same conditions, and the results showed that SWCNTs and MWCNTs increased performance of Ni^2+^ sorption compared to GAC, which can be explained by the fact that the CNTs possess increased surface when compared to the GAC. According to Yang et al. [57], adsorption of Ni^2+^ on MWCNTs increases with the pH. Sorption affinity between Zn^2+^ and CNT surface is stronger than sorption affinity between Ni^2+^ and CNT surface [53].

The results reported by Wang et al. [52] have shown a high adsorption of Pb^2+^ by MWCNTs. The Pb^2+^ adsorption on MWCNTs proved to be faster than AC and more, Pb^2+^ can be recycled under acidified MWCNTs.

Regarding the removal of Zn^2+^, purified SWCNT and MWCNT are better adsorbents than powder activated carbon (PAC) under the same initial concentration even if the PAC has a high surface than carbon nanotubes [59]. Sorption affinity between carbon nanotubes and zinc ions is higher than the affinity between Pb^2+^ and carbon nanotubes. According to Naghizadeh et al. [56], Cd^2+^ has a lower affinity for activated carbon to MWCNTs, and the capacities of Cd^2+^ adsorption on AC and MWCNTs are 2.9 mg/g and 4.1 mg/g, respectively. Comparing the removal of Pb^2+^, Cu^2+^ and Cd^2+^ by oxidized MWCNTs, the results indicate that Pb^2+^ has an increased adsorption capacity compared to Cd^2+^ and Cu^2+^. The adsorption of various HMIs on oxidized MWCNTs is ordered as follows: Cd^2+^ < Cu^2+^ < Ni^2+^ < Pb^2+^. The maximum adsorption capacities of Zn^2+^ on SWCNTs, MWCNTs and PAC, which were calculated using the Langmuir model, are 43.66, 32.68 and 13.41 mg/g [60], respectively.

#### 2.3.3. Low-Cost Bioadsorbents

The studies on low cost adsorbents have been focused on the separation of HMIs such as Pb^2+^, Zn^2+^, Ni^2+^ and Cd^2+^ from wastewaters. The results have shown that the percentages of HMIs removal depended on the amount of adsorbent, adsorbent concentration, pH and contact time.

Eggshells and their derivatives demonstrated a great potential for removing wastewater contaminated with nickel and silver ions. Comparing Eggshells and Eggshells with membrane, the values showed fast removal of Ni^2+^ and Ag for Eggshell membrane [61]. Moreover, according to Park et al. [62], the calcined eggshells showed a great potential for the adsorption of Cd^2+^ and Cr while the natural eggshells enhanced the adsorption of Pb^2+^. The removal efficiency for cadmium, zinc, chrome and lead was studied using chicken eggshells in aqueous solution [63] and wastewater [64]. These studies focused on the optimum pH, contact time, agitation speed and adsorbent dosages obtained from the experiment showed the ability of chicken eggshells for removing 99.7% of Cu^2+^ [63], 99% of Zn^2+^ [63], 85% of Cr, 82% of Pb^2+^, 86% Cd^2+^ [64] and 96.43% Fe [65]. These results can be explained by the increased number of active binding sites [63,66]. Eggshells are used also for wastewater treatment from battery productions due to their high removal efficiency and their natural characteristic as a pH adjuster [67]. These results also demonstrated that eggshells can be used pre-treatment of wastewaters due to the fact that they may remove a large quantity of heavy metals [62].

Agricultural Waste. Based on the previous study, agricultural water has the ability to remove heavy metals [68]. A good adsorbent with removal efficiency higher than 90% at pH 5 for heavy metals such as iron, copper and lead is Coconut husk [69,70]. More, a removal efficiency of 93% in 3 h of contact time, initial concentration of 25 mg/L of Zn^2+^ and pH 6.3 was obtained by the coconut shell after it was treated with acid and coated with chitosan. Another good adsorbent is coconut coir activated carbon, which was studied for copper and cadmium in water [71]. Rice husk is a low-cost adsorbent and contribute to economic yield [72]. Comparing polyaniline/rice husk with polyaniline and polyaniline/saw dust, polyaniline/rice husk showed a better efficiency removal of Cd^2+^ (93.08%) [73]. Comparing with values of the other adsorbents, the rice husk treated showed a higher adsorption capacity due to his surface area and pore size [74]. Nutshell has found to have ability to reduce heavy metals when modified cashew nuts. The maximum adsorption capacity of Cu^2+^ (406.6 mg/g), Cd^2+^ (436.7 mg/g), Zn^2+^ (455.7 mg/g) and Ni^2+^ (456.3 mg/g) [75]. Comparing chestnut shell pretreated with NaOH and pretreated with acid formaldehyde, the results showed that chestnut shell retreated with NaOH was more efficient in removing heavy metals [76,77,78]. Inyang et al. [79] reviewed the biochar as a low-cost adsorbent for the removal of heavy metal removal; they found that the sorption mechanisms depends on different biochars and HMI contaminants.

### 2.4. Membrane Filtration

The development of membranes and their filtration technologies has shown several advantages in the field of heavy metal removal due to their high efficiency, low space occupancy and simple design. The processes related to membrane filtration have been separated into reverse osmosis, ultrafiltration, electrodialysis and nanofiltration [70].

#### 2.4.1. Ultrafiltration (UF)

This method is used when faced with low transmembrane pressures in order to remove dissolved and colloidal materials. The pores used in this type of membrane are larger than the metal ions (as hydrated ions) and only low molecular weight complexes can pass unhindered through the membrane. In order to increase the removal efficiency, two technologies were developed such as micellar enhanced ultrafiltration (MEUF) and polymer enhanced ultrafiltration (PEUF) [80]. Table 4 presents the removal efficiency of heavy metals depending on the membrane used in each technology.

#### 2.4.2. Reverse Osmosis (RO)

In this method, a semi-permeable membrane is used, which separates the pollutant by allowing only the passage of the purified fluid. The method has the possibility to remove a large range of species, and it is used extensively in the desalination process [79]. In recent years, it has also been used in wastewater treatment and environmental engineering.

#### 2.4.3. Nanofiltration (NF)

This filtration method is a combination of UF and RO and incorporates advantages of both techniques when it is used in the removal of HMIs such as nickel, chromium, copper and arsenic from wastewater. Table 5 presents a comparison between the efficiencies of heavy metals removal by RO, NF and combined RO+NF. The most notable advantages of this method are simplicity, reliability and low energy consumption combined with a high rate of removal [39].

### 2.5. Coagulation and Flocculation

Coagulation and flocculation are processes used to remove the heavy metals from wastewaters, which also require sedimentation and filtration to collect the foam. Some of the most known coagulants used for wastewaters treatment are aluminum, ferrous sulfate and ferric chloride, which produce particulates and pollutants from wastewaters through particles charge neutralization. El Samrani et al. [94] obtained excellent results with respect to the removal of HMIs from waters by coagulation combining sewer overflow with ferric chloride and polyaluminum chloride (PAC). Suspended particles and hydrophobic colloids are the essential pollutants removed with this method. A new type of coagulant such as amphoteric polyelectrolyte (sodium xanthogenate with polyethyleneimine) was introduced in the method for removal of both soluble heavy metals and insoluble substance [95].

Flocculation refers to the use of polymers in order to form links between the flocs and formation of large aggregates. The suspended particles flocculate, and they can be detached by filtration, flotation or straining. Although many types of flocculants like PAC, polyacrylamide (PAM) or polyferric sulfate (PFS) are used for wastewaters treatment, it is impossible to use them for removal of heavy metals. Chang et al. investigated the use of a new kind of flocculant, i.e., macromolecule heavy metal flocculant (by reacting chitosan with mercaptoacetic acid) with good results regarding the turbidity removal as well as heavy metals removal from waters [96].

Even if coagulation and flocculation are efficiently and widely used techniques for heavy metals removal from waters, the use of nanostructures materials as coagulants/flocculants is not reported in the literature. A very important aspect in using nanomaterials in wastewater treatment is the small sludge quantity that results in comparison with classical coagulation/flocculation techniques.

### 2.6. Flotation

Flotation is used for isolating HMIs from the liquid phase using bubble attachment. The most used processes for water decontamination are dissolved air flotation (DAF), ion flotation and precipitation flotation. DAF allows bubbles to stick to particles, forming aggregates that ascent to the water due to their low density and are easily collected and removed as sludge [97]. Ion flotation surfactants are used for passing on the ionic metal species in hydrophobic wastewaters. The removal of cadmium, lead and copper from aqueous solutions was studied by Yuan et al. The results indicated removal efficiencies of more than 70% [98]. Precipitate flotation is also a flotation method based on precipitate formation which can be afterwards removed by attachment to air bubbles. The precipitation can occur by metal hydroxide formation or as salt [99].

## 3. Electrochemical Depolluting Treatments

Electrochemistry applied to remove HMIs from wastewaters has the advantage to recover the metals while treating the water from electroplating, food, oil, textile processing, etc. Among the most commonly used water purification technologies are electrodeposition, electrocoagulation (EC), electroflotation (EF) and electrooxidation (EO).

Besides corrosion, electrochemical industries produce effluents that are the main cause for water heavy metal pollution such as battery industry (Hg, Pb, Cd, etc.) or electroplating waste. The electrochemical recovery of metals from wastewaters has been adapted from the long-practiced electrometallurgy, in which metals were recovered from mine waters. Although the basic electrochemical mechanism based on cathodic deposition is very simple, i.e.,

$${\mathrm{Mn}}^{+}+{\mathrm{ne}}^{-}\to M,$$

the search for improvement in current efficiency and use of less expensive materials continues.

### 3.1. Electrodialysis (ED)

**Figure 3:**
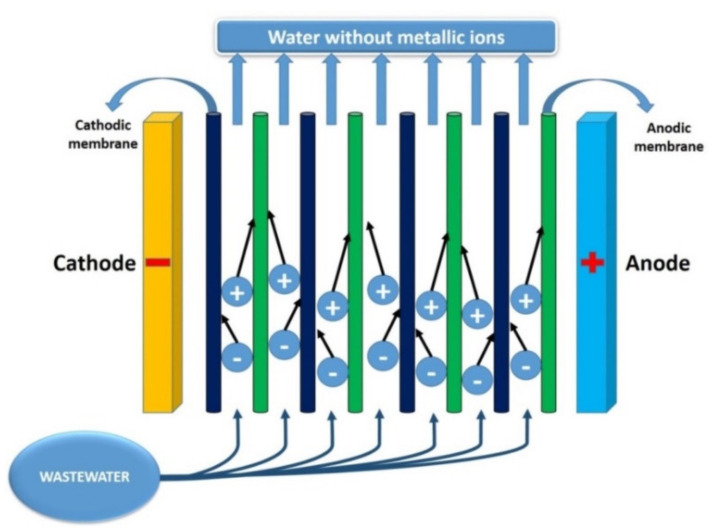
Illustration of the electrodialysis operation principles. The membranes have anionic or cationic property. When the mixture passes through the membranes, the ions are moved to the direction of the anode or cathode.

This method involves the use of a separation membrane and electric potential in order to remove pollutants from a solution as it passes through the system. Figure 3 illustrates the principles of electrodialysis. Separation membranes exhibit anionic or cationic characteristics. As the solution passes through the membranes, the ions are attracted towards the anode or cathode [100].

Tzanetakis et. al. [101] have performed experiments with electrodialysis in order to remove Ni(II) and Co(II) ions from synthetic solutions and compared the capabilities of two cation-exchange membranes made from perfluorosulfonic Nafion 117 and sulfonated polyvinyldifluoride membrane (SPVDF). The sulfonated PVDF membrane is a low-cost membrane that showed chemical, thermal and mechanical behavior was similar to the Nafion 117. Moreover, separation of elements was possible when a mixture of nickel and cobalt was used. The separation took place in the presence of ethylenediaminetetra-acetic acid (EDTA) when the [Ni-EDTA]-2 complex was preferentially formed and retained, while the cobalt ions passed through the SPVDF membrane [101].

At concentrations of more than 500 ppm, the separation performance is reduced considerably. In order to increase the efficiency, high ion exchange capacity is required for the exchange membranes. Jakobsen et al. [102] examined the removal of cadmium from wastewater by exposing the solution to an electrical DC field. The proportion of liquid solution to solid sludge was 1.4 and 2. The sludge was mixed with citric acid, HNO_3_ or distilled water, with a removal efficiency for each of the experiments of 70%, 67% and 69%, respectively.

### 3.2. Electroflotation (EF)

This technique is commonly used in the wastewaters treatment and has applications in the removal of heavy metal ions from industrial wastewaters. In the electroflotation process, pollutants, iron or solid particles adhere to tiny bubbles of H_2_ or O_2_ that were formed on the electrodes of the floatation cell and float to the surface. Some additives, aluminum or ferric salts are commonly brought into the waters for diminishing the suspended particles and complete a natural separation. Belkacem et al. [103] investigated the removal of iron, nickel, copper, zinc, lead and cadmium using aluminum electrodes. The removal efficiencies reached 99%. When electroflotation is used in aqueous media, water electrolysis forms very fine bubbles of hydrogen and oxygen toward the reactions:

$$\mathrm{Cathode}:\;2H_{2}O\;+\;2e^{-}\;↔\;2{\mathrm{OH}}^{-}\;+\;H_{2}(g)$$

$$\mathrm{Anode}:\;H_{2}O\;↔\;2H^{+}\;+\;1/2O_{2}\left(g\right)\;+\;2e^{-}$$

The gas evolution process can be split in three physical processes: nucleation, growth and detachment. The bubbles nucleation takes place at electrode surfaces from highly supersaturated solutions [104]. They are removed from the electrode by forces of buoyancy or liquid shearing forces that pull the bubbles away. In electroflotation, the bubbles size depends mainly on the nature of the electrode material and its location in the electromotive series. The solution pH influences the bubble size, i.e., smaller hydrogen bubbles are formed in neutral or alkaline media in comparison with the acidic media. Unlike hydrogen, oxygen bubbles achieve a minimum size in acidic media, increasing in diameter as the pH increases. The detachment size of the bubbles is also influenced by the shape of the electrode surface. There have been conflicting investigations regarding the effect of current density on bubble size. An increased current density was reported by Mansour et al. [105] by increasing the diameter of hydrogen bubbles, which was explained by the coalescence of the bubbles at high current densities. On the other hand, Kektar and Burns et al. [106,107] have concluded the opposite.

### 3.3. Electrocoagulation (EC)

In electrocoagulation, electrical current is used in an electrochemical setting to remove metals from water. It is usually combined with other wastewater treatments to remove pollutants, especially heavy metals, from various industrial wastewaters such as electroplating and food industry. Unlike the traditional coagulation techniques, electrocoagulation uses an electrical current to remove pollutants, which proved to be beneficial especially in the case of heavy metal. Moreover, the electricity required to operate the electrocoagulation process may be provided by renewable energy sources such as solar, wind, geothermal, biogas and small hydro. Electrocoagulation is very similar to chemical coagulation. In electrocoagulation, the coagulants are required to be immersed electrochemically in the polluted waters using aluminum or iron sacrificial anodes. Pollutants are then kept into the solution through electrical charges. When these ions interact with ions charged with reversed electrical charges by applying certain electrocoagulation conditions, they lose stability and precipitate in a constant configuration.

The electrocoagulation process offers many advantages in comparison with the traditional coagulation technique [108]. The main advantages include no additional chemicals introduced in the wastewaters before treatment, the electrocoagulation process is straightforward, pollutants can be easily neutralized, and there is no secondary pollution. The equipment required for operating the electrocoagulation technology is simple and easy to use. The costs of operation are low, and the process automatization is possible. Moreover, the smallest colloidal particles can be efficiently removed by electrocoagulation due to the fact that they move faster under the applied electric field, which facilitates the agglomeration.

For the electrocoagulation process to take place, there are three successive steps necessary, as follows:

- Formation of coagulants by electrolytic oxidation of the sacrificial anode electrodes.
- Destabilization of the contaminants, particulate suspension and breaking of emulsions.
- Aggregation of the destabilized phases to form flocs.

The flocs formed during the process are large and stable and can be separated by simple filtration techniques. The flocs are carried to the top of the solution by the gas bubbles formed during water electrolysis where they are concentrated, collected and then removed. Compared to other treatments, the flocks from the electrocoagulation process can be reused with lower costs as result of lower solids content. From the electrocoagulation process results low quantity sludge that can be easily processed and dewatered because of its composition, i.e., metallic oxides and hydroxides. Electrocoagulation process can be also used in rural areas where electricity is not available or with limited access to electricity, remote areas or isolated areas, by attaching a solar panel or other renewables to the water treatment system [109]. Table 6 shows the removal efficiency of several heavy metal ions, along with the electrode material and initial concentration of HMIs in solution.

**Figure 4:**
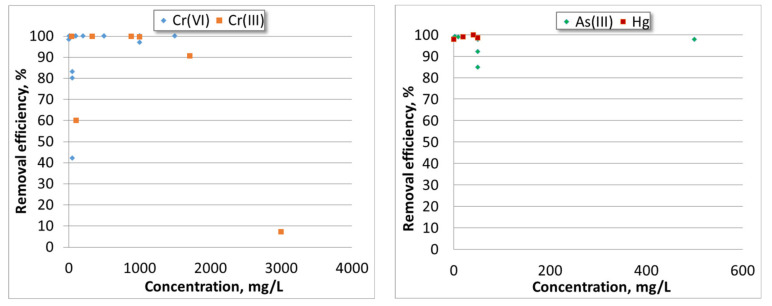
Removal efficiency of Cr (III), Cr (VI), As (III) and Hg function of the initial concentration of ions in the water.

A comprehensive review on electrocoagulation was published by Al-Qodah et al. [118]. The removal efficiency depends on several factors such as the initial concentration in the water, the electrode material and the pH of the water. Figure 4 presents the removal efficiency of Cr(III), Cr(VI), As(III) and Hg as a function of the initial concentration of HMIs in water (adapted from [109,119]).

The arrangement of electrodes in the electrocoagulation process and the electrode material are very important with respect to the cost analysis. The treatment of textile water was investigated by Kobya et al. [115] using three different ways of electron arrangement:

- Monopolar electrodes in parallel connection, where current is divided between all electrodes;
- Monopolar electrodes in a serial connections system, where every couple of sacrificial electrodes are connected with each other;
- Bipolar electrode in serial connections where there is not any electrical connection between the inner electrodes because the outer electrodes are linked to power.

The spacing between electrodes is very important for the removal of pollutants. In order to diminish the energy consumption, it is indicated to use larger spaces between electrodes for effluent with high conductivity treatment. Otherwise, in case of effluents with low conductivity, the energy consumption can be diminished by smaller spacing between electrodes.

**Figure 5:**
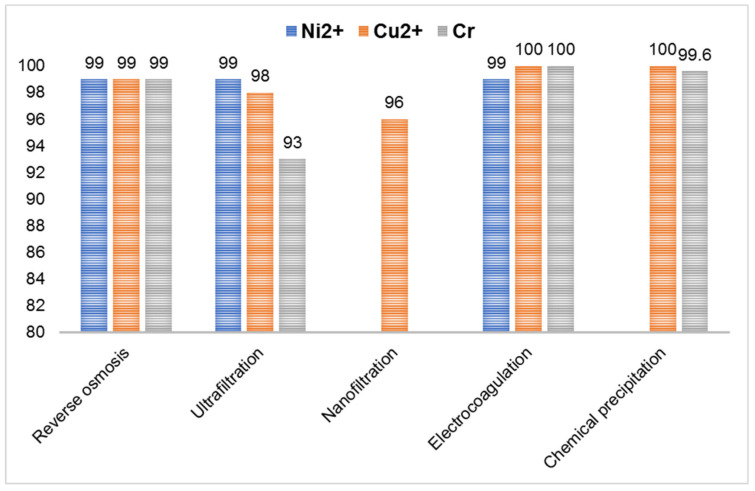
Level of removal efficiency for Ni^2+^, Cu^2+^ and Cr as a function of the method of their removal (adapted from [109]).

The process of electro-coagulation is extensively used for removing solids in suspension, dissolved metals, tannins and dyes. These contaminants that are present in wastewater are maintained in the solution through electrical charges. Table 7 shows several water treatment methods to remove heavy metal ions. Saturating the water with ions of opposite electrical charge to that of the heavy metal ions leads to the destabilization of the latter, and they start to precipitate into a stable form, facilitating the removal from the water. A variety of pollutants can be eliminated through electrocoagulation such as arsenic [120], strontium and cesium [121], phosphate [122], sulfide, sulfate and sulfite [119], boron [123], fluoride [124], nitrate [113], chromium [125], cadmium [114], zinc [115], nickel [126], mercury [127], and cobalt [128] as well as oil [129], chemical oxygen demand [130], color [131] and organic substances [132]. The removal efficiency of HMIs by different methods is presented in Figure 5, where electrocoagulation has a better efficiency for Cu and Cr ions removal than reverse osmosis and better removal efficiency for Cr than chemical precipitation.

The following considerations are the main factors affecting the efficiency of the electrocoagulation process:

**Current density.** In terms of current density in the electrocoagulation system, it is the main factor that “decides” the quantity of Al^3+^ or Fe^2+^ ions discharged from the electrodes. Each element has a specific electrochemical equivalent mass: 335.6 mg/(Ah) for Al and 1041 mg/(Ah) for Fe. Using a higher current leads to the possibility of using a smaller electrocoagulation unit, but this current also heats up the water and can result in a waste of electrical energy. Moreover, a current that exceeds the necessary parameters results in a decrease of process efficiency. The current density suggested for a long operation of the electrocoagulation system without maintenance is 20–25 A/m^2^. If the electrodes are periodically cleaned, this value can change depending on the operating factors.

**Addition of NaCl.** Generally, table salt is used so that the water conductivity can be increased to facilitate treatment. Usually, in wastewater, there are certain anions present, such as HCO_3_^−^ and SO_4_^2−^, which have the added disadvantage of precipitating Ca^2+^ or Mg^2+^ ions. These ions adhere to the surface of the electrodes and insulate them, resulting in an increase in potential and a decrease of current efficiency. Chloride ions are used to inhibit these adverse effects, and it is usually recommended that there should be approximately 20% Cl^-^ ions present in the water in order for the electrocoagulation process to proceed normally.

**pH Effect.** pH can affect the electrocoagulation of water or wastewater though and increase or decrease of current efficiency as well as its influence on the solubility of metal hydroxides. In the case of aluminum, the efficiency of removal of ions through current density is higher in acidic or alkaline conditions, while most pollutants react more positively to a pH of approximately 7.

**Temperature.** Increasing the temperature usually results in a modification in pore size of Al(OH)_3_ gel. These pores reduce in size, and the generated flocs tend to deposit themselves on the surface of the electrode. Similar to the current efficiency, the power consumption also gives a maximum at slightly lower value of temperature, 35 °C, for treating oil-containing wastewater [47]. This process can be explained due to the opposite effect that temperature has on water, resulting in a higher conductivity at higher temperature, meaning lower energy consumption.

**Power supply.** The passing of a current inside an electrochemical reactor can only be done by overcoming different types of potentials: anode overpotential, ohmic potential drop, cathode overpotential and equilibrium potential difference [20]. The anode overpotential is comprised of concentration overpotential as well as activation overpotential, and it can also be influenced by a passive overpotential of the passive film on the surface of the anode. The cathode overpotential is determined by concentration overpotential and activation overpotential.

The main advantages for using electrocoagulation processes in the treatment of water as compared to coagulation process are the following: (I) organic matter is separated faster and effective, (II) there is no need for pH control, (III) the small amount of chemicals used in the process, (IV) a small amount of sludge is produced and (V) low operation costs.

Among disadvantages, the most important are (I) high concentrations of iron and aluminum ions in the effluent that have to be removed and (II) that the hydroxide does not present an appropriate grain size to ensure its precipitation. This results in difficulty to ensure separation, which also results in an increase in voltage in order to overcome the lack of hydraulic properties of the material.

### 3.4. Electrochemical Technologies in Wastewater Treatment

In electrochemical processes, the most important factors are potential, current, current density, *i_a_* (current per electrode area), current efficiency, *CE* (i.e., how much current is consumed to produce a certain product vs total consumption). For metal recovery, the electrochemical reactors are also defined by their performance given by the space–time yield of the reactor, *Y_ST_*, i.e., the amount of product produced by the reactor volume in unit time:

$$Y_{\mathrm{ST}}\;=\;\left(i_{a}M/1000zF\right)\;\times \;CE.$$

Electrochemical deposition is effective in recovery heavy metals from wastewater streams [100]. The main advantage of using electrochemical treatment of clean water is that it does not introduce additional chemicals. Moreover, heavy metal ions can be removed selectively by electrochemical treatment.

**Figure 6:**
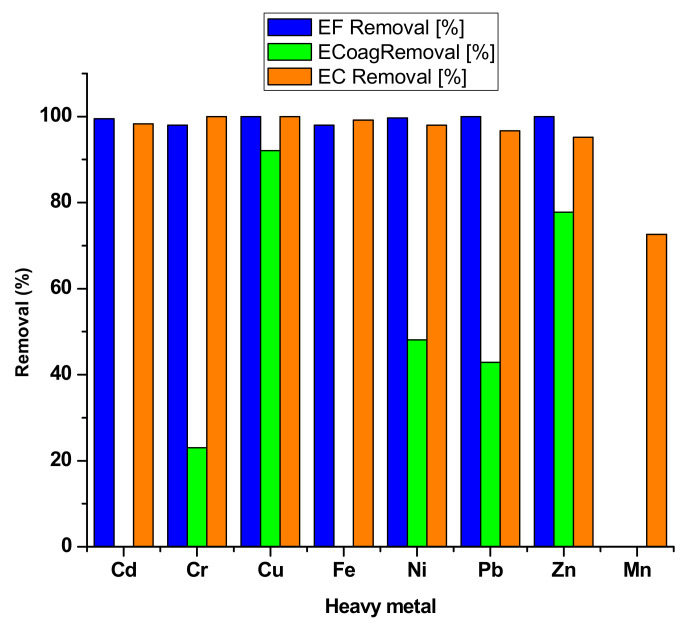
The efficiency of HMIs removal by different methods such as electrocoagulation (ECoag), electroflotation (EF) and electrochemical removal (EC) (adapted from [118]).

Figure 6 presents a comparative analysis of the removal efficiency for heavy metals as a function of the method of their removal. Among the electrochemical methods, electroflotation shows consistent good removal efficiencies for all the heavy metals tested, followed by electrochemical removal, while electrocoagulation shows poor efficiencies (lower than 50% for Cr, Ni and Pb).

## 4. Electrochemical Detection and Removal of Heavy Metals

**Figure 7:**
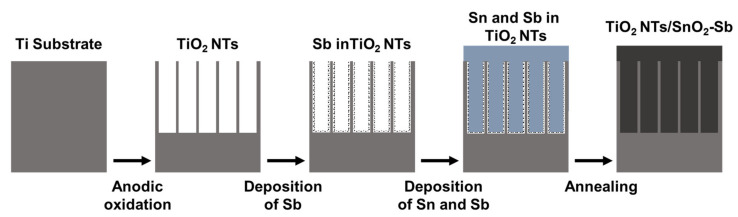
Schematic illustration of the vertically aligned nanotube electrodes.

Because of ineffective elimination of HMIs to accepted values by conventional treatments [26,27], new electrode materials have been developed. For instance, titanium dioxide (TiO_2_) on metallic titanium has been used for many photoelectrochemical oxidations processes. However, changing the TiO_2_ morphology from film to hierarchical TiO_2_ nanotubes (TiO_2_-NTs) improves electron percolation at the TiO_2_ interface due to the enlarged surface area associated with the high structural organization, which increases the reactions at surface/electrolyte interface and reduces the recombination levels of electron/hole [138,139]. Because TiO_2_ nanotubes have low toxicity, good stability and high efficiency, TiO_2_-NTs have been an ideal material for organic pollutants removal applications. Figure 7 shows a self-explanatory illustration of the formation process of TiO_2_-NTs/SnO_2_-Sb electrodes.

The use of mixed metal oxide (MMO) anodes has been extended to anode material for electrochemical oxidation of certain pollutants that are very difficult, if not impossible, to remove from aqueous environment. These electrodes with high oxygen evolution potentials are used to remove difficult organic pollutants in water through electrochemical oxidation, depending of the mechanisms involved (i.e., by direct oxidation or mediated oxidation with the MMO anodes) including dyes, pesticides and herbicides, phenolic compounds, pharmaceuticals, antibiotics and hormones, plasticizers, perfluorinated chemicals, surfactants and derivatives, chelating agents and microcystin toxins [140]. Since major reactions take place in the MMO anode surface, increasing the anode surface can be achieved by template synthesis. Examples include vertically aligned TiO_2_-NTs used as template for deposition of PbO_2_ [141], SnO_2_ [142] and WO_3_ [138].

Carbon nanotubes (CNTs) based electrodes are another example of electrode developed to address the challenges encountered in certain pharmaceutical waters. Modified multiwall carbon nanotubes (MWCNTs) electrode doped with Ce was used to remove ceftazidime by electro-oxidation, which is an aqueous antibiotic. Cerium doping of the MWCNTs electrode films alters the morphology of the electrode surface and improves the oxidation ability of the electrode [143]. Cyclic voltammetry showed that the modified MWCNTs electrodes present a wider oxidation peak and generally display stronger capacities of generating reactive groups. In addition, the oxidation peak potential of the modified MWCNTs electrodes was smaller than that of the unmodified MWCNTs electrodes. The electrochemical removal of ceftazidime in an aqueous solution on the modified MWCNTs electrodes showed that the removal efficiency of ceftazidime with an initial concentration of 1mg/L was approximately 100% after 60 min electrolysis in a 1 g/L Na_2_SO_4_ electrolyte with a current density of 3mA/cm^2^ and an electrode spacing of 1 cm [143].

There are a number of methods for growing nanostructures but for detection, the surface area of the nanostructures should be very large and uniform. The most useful method to obtain ordered nanostructures with high aspect ratio is template synthesis (TSy), which provides quantifiable results.

### 4.1. Methods to Obtain Nanostructured Electrodes

Nanostructured electrodes can be obtained with or without templates. However, nanostructure array with high aspect ratios (L/d > 20) are typically obtained in porous templates with transversal pores. For this particular nanotube and nanowire arrays, several methods have been developed depending on the system to be obtained and the properties of the template. Electroless and electrochemical deposition are the main methods developed for obtaining nanotube or nanowire arrays, which also allows the seamless integration of these nanostructure arrays into devices such as sensors.

#### 4.1.1. Electroless Deposition 

Electroless process was developed based on the reduction process of metallic ions from solution and oxidation of a compound from solution, as reducing agent, followed by film deposition as a result of the internal current produced in the process. According to Sudagar et al. [144], electroless process is an autocatalytic method which appears due the presence of a cation of the metal reduced by the electrons presented onto the metal surface of a substrate or a catalyst responsible for beginning deposition process.

Although nanowires can be obtained without a template, it is necessary to use a template when nanowires with high aspect ratios are needed. There are a few materials used as templates that have transversal pores, as follows: (i) insulating materials such as alumina and (ii) track-etched polymers like polycarbonate (PCTE) and polyethylenterephtalate (PET). In the electroless deposition process, gold or other metal deposition takes place on the entire surface of the membrane used as template including the inside walls of the pores.

Polymeric membranes: Au electroless deposition is typically obtained using PCTE filtration membranes with various diameters, from 30 to 200 nm. In this method, commercial gold electroless plating solution (Oromerse Part B, Technic Inc.) is diluted 40 times with water prior to use to obtain a typical composition of 7.9 × 10^−3^ M Na_3_Au(SO_3_)_2_ and 0.127M Na_2_SO_3_. Electroless deposition procedure starts with immersing the PCTE template membrane for 2 h in methanol, followed by sensitization with SnCl_2_ solution and trifluoroacetic acid in 50:50 methanol–water for 45 min. After membrane sensitization, a solution of Ag[(NH_3_)_2_]NO_3_ is added. Then, the membrane is immersed in an Au plating bath containing formaldehyde. Another option is to add formaldehyde after soaking of membrane into gold electroless bath at about −4 °C. Electroless deposition is extended for different times, and finally, the membrane is washed with water, immersed in HNO_3_ for 12 h, washed again with water and dried at room temperature [145].

Martin et al. [146], who first introduced the electroless metal deposition process for nanoporous PCTE templates, have shown that this process requires a chemical reducing agent in order to deposit, as slow as possible, a metal from solution onto a surface that is not electronically conductive. The presence a catalyst can accelerate the metal reduction rate.

**Figure 8:**
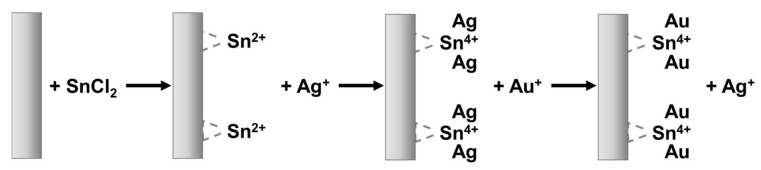
Illustration of the electroless deposition process.

Figure 8 presents an illustration of the Au electroless deposition process. The PCTE membrane is first sensitized by immersing it into tin chloride solution SnCl_2_ and trifluoroacetic acid, using a mixture of 50/50 methanol/water as solvent. After washing with methanol, Sn^2+^ attach to the surface of the membrane, which was covered by a thin layer of poly(viny1pyrrolidone) (PVP) to improve hydrophilicity. Then, the membrane is activated in an aqueous solution of ammoniac and AgNO_3_. A redox reaction takes place with the oxidation of Sn (II) to Sn (IV) and the reduction of Ag (I) to Ag (0). Good results on the activation of Sn (II) and reduction of Ag (I) were obtained using glass substrate.

After Ag coating, the membrane is immersed into Au plating solution, which consists of Na_3_Au(SO_3_)_2_, Na_2_SO_3_ and formaldehyde. In this step, Ag is replaced by Au and the membrane becomes coated by Au. In the case of gold deposition, it is essential that the formation of Au nuclei takes place before the growth process begins, as these Au nuclei act as “catalytic sites” for the next Au deposition, when formaldehyde is added to reduce Au and increase the Au deposition rate. All Au ions are reduced to Au atoms by formaldehyde, which acts as reducing agent, according to the following reaction:

$$2{\mathrm{Au}}^{+}\;+\;\mathrm{HCHO}\;+\;3{\mathrm{OH}}^{-}\to {\mathrm{HCOO}}^{-}\;+\;2H_{2}O\;+\;2\mathrm{Au}$$

As the Au coating advance and the entire pore surface is coated, Au nanotubes form. A rapid growing of the nuclei can lead to pore clogging pretty fast, which will stop the deposition process inside that pore. Nanowires can also be obtained by this method at longer deposition time, in about 24 h [146,147].

Anodized Aluminum membrane (AAO). Another template used for nanowires synthesis is the porous aluminum oxide (anodic aluminum oxide, AAO) membrane, which exhibits better stability and chemical inertia compared to PCTE [148]. AAO templates are obtained using an aluminum foil (99.99%-Merck). First, the foil is chemically treated under normal conditions using NaOH and washed with distilled water and acetone. To obtain a uniform AAO array template, the second step consists of surface anodization by applying a constant voltage and a mixture of acids [149].

Because the AAO membrane is not conductive, electroless deposition technique was used to obtain ordered Cu, Ni and Co nanotubes inside AAO membrane template [150]. The nanotube dimensions were imposed by the membrane size, i.e., the outside diameter of the nanotubes was given by the pore size, while the length of the nanotubes was equal to the thickness of the template. The electroless deposition is similar for all metals deposited and usually starts with a redox reaction in SnCl_2_ and HCl, followed by an activation process [146,150]. Due to the advantage regarding the obtaining of nanowires without electronically conductive surface, electroless deposition could offer a good alternative for nanowires synthesis. For short time electroless deposition, nanotubes can be obtained while nanowires are obtained at longer deposition times. Besides metal nanostructures, metallic alloys can be also obtained. Yuan et al. [151] obtained Co-P nanowires with magnetic properties in AAO template using cobalt-plating bath made using cobalt chloride, sodium hypophosphite, sodium citrate and ammonium chloride [151]. Several metallic nanowires were produced by electroless deposition, which will be briefly presented below.

Au nanowires (Au NWs) with strong mechanical resistance, stability, and minimal defects were obtained using cyanide-complexed gold solution [146,152,153,154]. An important aspect of this method is the environmental impact and the high toxicity of gold cyanide, as the most commonly electrolyte used in this process [155,156,157]. Although the cyanide solution prolongs the life of the electroplating bath, new alternative methods appeared lately to prevent this disadvantage. For example, gold sulfite [158] and hydrogen tetrachloroaurate (HAuCl_4_) have been the subject of several reports on the synthesis of Au NWs by electrodeposition or electroless deposition [159,160,161]. Wang et al. [161] synthesized Au NWs at different temperatures in one-step process using ethanol as a reducing agent for HAuCl_4_.

According to Kan et al. [160], Au NWs could be obtained using mesoporous silica by immersing the silica in HAuCl_4_ solution, followed by drying and heating at about 300 °C, without any reduction treatment. An explanation of the mechanism of Au nanowires formation could be diffusion of Au atoms along of silica-controlled pores combined with a nucleation process that takes place at low rates.

An interesting method for Au NWs growth was developed by Kim et al. [162]. In this method, Au seeds nanoparticles were prepared using NaBH_4_ to reduce HAuCl_4_. The Au nanoparticles generated an in situ process of autocatalytic reduction of Au (I), thus expanding the seed nanoparticles. The synthesis of Au NWs was performed by adding these Au nanoparticles to a solution mixture containing gold precursor HAuCl_4_, ascorbic acid that was used for the reduction of Au (III) to Au (I), and a structural directing agent CTAB (hexadecyl-trimethylammonium bromide) [162].

Ag Nanowires. A facile electroless synthesis for silver nanowires (Ag NWs) using polyvinylpyrrolidone (PVP) surfactants can be developed on different substrates such as polyethylene terephthalate (PET) or glass at room temperature. The PVP molecules around Ag NWs offer good protection and stability of these NWs [163].

Promising results were obtained with ion-track etched mica templates used for growing of metal thin films. In the rhombohedral pores of the template, distinctive nanowires can be deposited having an aspect ratio of up to 70. Based on Sn(II)/Ag(I) redox system, Ag nanoparticle seeds could be accumulated on the template surface. Moreover, this method worked for Pt NWs too [164].

Ni Nanowires. Besides, Au and Ag, Ni NWs can be obtained using chemical reagents such as source of Ni (NiCl_2_·6H_2_O), reducing agent (hydrazine monohydrate: N_2_H_4_·H_2_O), solvent as ethylene glycol (EG), complexing agent (chloroplatinic acid hexahydrate: H_2_PtCl_6_·6H_2_O) and nucleating agent (trisodium citrate dehydrate: Na_3_C_6_H_5_O_7_·2H_2_O). Moreover, sodium hydroxide (NaOH) was used. Reaction took place under temperature in a water bath where two parallel neodymium magnets separated were placed for assuring the direction of Ni NWs formation. After the reaction, nickel nanowires were washed with ethanol. Nanowires made of iron group metals (Fe, Co, Ni) display a strong potential for certain applications from catalytic materials to magnetic materials because of their magnetic shape anisotropy [165].

Co Nanowires. Metallic Co NWs were prepared by electroless deposition at room temperature using propylene glycol under external magnetic field. The Co NWs obtained had a mean diameter of about 190 nm and lengths up to 160 µm Co. Due the strong magnetic interactions that appeared in the direction of the applied magnetic field, the Co nanoparticles were assembled [166].

#### 4.1.2. Electrochemical Template Synthesis of Nanostructures

Electrochemical nanotechnology (nanoelectrochemistry) has become increasingly important due to certain unique properties observed at nanoscale. A decrease in the size of the electrode causes changes in the form of the diffusion layer from linear to spherical. The diffusion layer is formed as oxidized species are consumed at the electrode surface. This results in a concentration gradient between bulk concentration and depleted regions that leads to a decrease of the diffusion limited current during long operations. The behavior of nanoelectrodes during different operation times has led to the consideration of the diffusion mechanic as being planar during short usage and spherical during long operations. On the other hand, for electrodes at the nanoscale, diffusion phenomena are very fast so that normally mass transfer limitations are negligible, and surface kinetics control the deposition process. For multiscale nanostructures such as nanotubes, nanofibers, and nanocables, it is important to know which characteristic length scale, nm or µm, governs the deposition process [167,168,169]. For the µm scale, diffusion limitations can be important if the surface deposition processes are relatively fast.

Electrochemical template synthesis is mainly used to obtain arrays of nanoelectrodes. The advantages of using nanoelectrode arrays are generally due to their small size that results in maintaining a very steady current and a strong current/unit area. Moreover, this system ensures a small potential drop that makes it favorable for use in measurements in solutions with low concentrations of electrolyte. Another important advantage is that the array can function at room temperature in order to perform electrodeposition processes. This inhibits interdiffusion between adjacent layers in the deposition of multilayered nano-sized materials.

**Figure 9:**
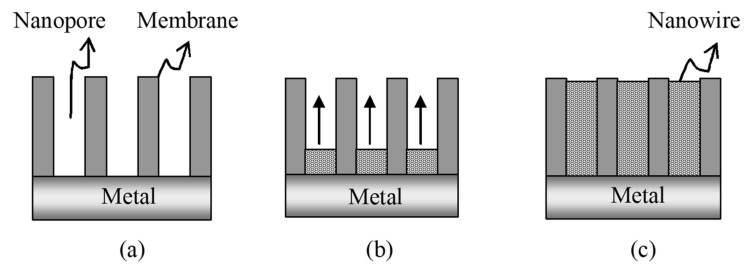
(**a**) Nanoporous membrane that has one side coated with a metallic film. (**b**) Electrochemical deposition through the nanopores (the arrows indicate the growth direction of nanowires inside pores). (**c**) Nanowires obtained inside nanoporous membranes.

Electrochemical template synthesis of a material within the pores (Figure 9) begins by coating one face of the template with a metal film (usually via either ion sputtering or thermal evaporation) and using this metal film as a cathode for electroplating [170,171]. This method has been used to prepare a variety of metal nanowires in both track-etch and alumina templates. Additionally, the potentiostatic growth of the nanowire array through the etch tracks can be performed under convection control in an electrochemical jet cell [172]. The lengths of the nanowires can be controlled by varying the deposition time. This ability to control the length or aspect ratio (length to diameter) of the metal nanowires is especially important in optical applications because the optical properties depend on the aspect ratio.

This method can also be used to prepare hollow metal tubules [173]. To obtain tubules, the pore walls must be first chemically modified (i.e., molecular anchor must be applied) so that the electrodeposited metal preferentially deposits on the pore wall. For example, gold tubules have been prepared by attaching a cyanosilane to the walls of the alumina template membrane prior to metal depositions [174]. This method has the added benefit of being used to tailor the pore walls in alumina membranes for different applications using silanes that are commercially available.

**Figure 10:**
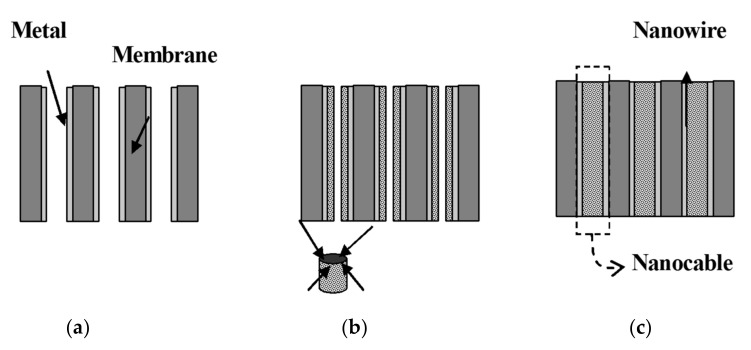
(**a**) Metallic nanotubes obtained inside the polycarbonate (PCTE) membrane, as electric conductive base for the electrochemical deposition of further materials. (**b**) Pores are filled by radial electrochemical deposition (arrows show the growth direction inside the pore). (**c**) Nanocable structures (i.e., filled nanotubes) formed in the PCTE membranes.

The most important aspect in the electrochemical template synthesis is the conductive surface used as electrode, which can be formed on a given template in two ways as depicted in Figure 9 and Figure 10. When the conductive layer is deposited on one face of the membrane (Figure 9), the nanowires can grow through the pores of the membrane, which acts as a template. The main advantage of this method is the possibility of growing nanowire arrays directly into a device such as sensor, thus increasing the manufacturing process efficiency and reducing the costs.

Figure 9 illustrates the formation of an array of nanoelectrodes in a membrane, where the electrode material was applied on one of the two facets of the membrane. Another way to make the surface conductive for further electrochemical deposition is to use electroless deposition, as illustrated in Figure 10, where the conductive layer is deposited on the walls of the pores, creating nanotube structures. Ku et al. [175] obtained Au/Te nanocable-like structures using electroless deposition of Au to coat the inner wall of the pores and then an electrochemical process to grow Te inside the Au nanotube.

#### 4.1.3. Electrochemical Template Synthesis for Nanostructure Arrays

Various composite nanostructures can be fabricated using electrochemical methods such as template synthesis. It is well-known that electrodeposition in AAO results in nanowire arrays of Fe [176,177], Co [166,173,178] and Ni [148,178,179,180] and their alloys. Composite materials composed of a variety of conductors, insulators, semiconductors and photoconductors were obtained. The number of different components that each composite can accommodate depends only on the template (initial diameter of the template pore, the thickness of the membrane) and the deposition technique (e.g., rate of material deposition, temperature).

**Nanowires and Nanotubes Array**: Nanowire and nanotube structures with monodisperse diameters and lengths can be fabricated. Generally, 1D nanostructures show superior properties and functionality when compared to their larger forms. Small structures with large surface area and nanoscale quantum confinement effects have unique chemical, optical and electronic properties compared to bulk materials. These are the simplest structures that can be obtained inside the pores, yet they are still complex. The template method can be used for the synthesis of metals, alloys, semiconductors or composites in space confined volumes of the membrane pores.

In membranes with transverse pores, hollow or filled cylindrical structures can be created, depending on the synthesis method. An important feature of this method is the possibility to tailor the size of nanotubes and nanowires for specific applications. Moreover, template synthesis for metal nanostructure fabrication is an attractive alternative solution to overcome nanofibrils fabrication using lithographic methods. Using template strategy combined with electrodeposition technique, nanometer-sized metallic wires, super conducting nanowires and magnetic multilayers can be fabricated [175], which exhibit physical properties different from those found in the bulk.

**Gold.** Gold NTs and NWs are generally obtained by electrochemical plating or electroless displacement. Because electroless deposition results in a more uniform Au deposition [146], it is the preferred method for Au nanotubes while electrochemical deposition is the preferred technique for nanowire template synthesis [147,181,182,183,184].

Among the earliest applications of the TSy of Au nanostructures was preparing ensembles of nanoelectrodes. Such electrodes are simply obtained by Au deposition in PCTE membranes by either electroless or electrochemical deposition. The electrodes can be in the form of nanoelectrode ensembles or arrays of gold nanotubes. Nanoelectrode ensembles are simply Au nanodiscs (i.e., the active electrode area) ensemble into the membrane [147]. The electrochemical signal of the cyclic voltammogram at these nanoelectrode ensembles is very strong, making the electrodes useful in ultra-trace detection of electroactive species.

Electrode ensembles of nanotubular Au obtained by electroless deposition provide a novel approach as glucose biosensors. Glucose oxidase (Gox) has been studied through immobilization onto preformed monolayers (mercaptoethylamine or mercaptopropionic acid) on electroless gold by cross-linking with glutaraldehyde [185,186]. Under optimized conditions, the detection limit was 2 × 10^−4^ M.

Arrays of Au nanotubes can be obtained after the polycarbonate membrane is removed to obtain freestanding nanowires. With their large electroactive surface area, arrays of Au nanotubes provide additional advantages in electroanalytical applications. One of the applications is as a novel template for making glucose sensors with a large amount of enzyme electrochemical entrapped into the ultra-thin nanotube array. Glucose sensors based on the traditional electrodes (such as platinum, gold or glassy carbon) monitor glucose by detecting hydrogen peroxide. On arrays of Au nanotubes, glucose sensors show a high sensitivity and high selectivity H_2_O_2_ detection. The sensitivity is amongst the highest values reported in the literature for comparable biosensor systems [185,186,187]. Another application of the arrays of single crystalline gold nanowires is in field emission [188].

Electrochemical deposition offers the possibility to create single crystal Au nanowires with an average aspect ratio of 100 in the pores of PCTE membranes [189,190]. The potentiostatic EC-TSy of Au nanowires results in “cigar like” structures [191]. The wire diameter, which should reflect the pore diameter, varies in the cross section: The wire’s diameter is noticeably smaller at both ends than in the middle. Morphology studies performed with transmission electron microscope (TEM) indicate that the surfaces of nanowires obtained by electrochemical deposition are smooth and exhibit single crystal structure [192]. Additional high-resolution TEM and electron diffraction [154,193,194,195,196,197] have shown that the most prevalent planar defects in face-centered cubic metallic nanocrystals is twinning. In single-crystal Au nanowires, microtwins were seen to depend on the growth direction of nanowires, i.e., twinning was observed in single-crystal NWs with a [111] growth orientation, but not in NWs that had a growth direction in [100] and [110]. Metallic characteristics as shown by the I–V curves of individual nanowire were revealed by AFM measurements performed with a conductive tip operating in contact mode at room temperature in air [192].

**Silver.** Single crystal Ag nanowires have been fabricated by electrochemical [189,190] or electroless deposition [103,126] in the pores of PCTE membranes. However, Ag NWs with high aspect ratio of 100 have been obtained via electrochemical deposition [189,190]. The electroless deposition is an inexpensive solution to the challenges posed by Ag NWs fabrication for various applications in optics, electronics and biological fields. Besides the classic electroless deposition of metals, a modified method can be used to obtain single crystal nanowires [198]. The initiation layer for silver crystal growth is represented by a gold film grown on one side of the membrane pores and the pores act as guiding funnels for growing the silver into a cylindrical nanostructure.

**Cobalt**. The electrodeposition of Co as well as other ferromagnetic metals (e.g., Fe, and Ni) in membrane nanopores results in a high-density surface distribution of ferromagnetic columns isolated by membrane, and high perpendicular anisotropy in the magnetic field. These unique properties induced by the size, shape and distribution of the ferromagnetic nanostructures are beneficial for enhancing the recording density media.

Cobalt has a hexagonal closed packed (hcp) crystal structure with the c axis as the easy axis of magnetization. Co NWs have enhanced magnetic coercivity [199]. Additionally, resonance phenomena have been observed for Co nanowires array [200,201].

Co nanowire arrays are generally obtained by electrochemical deposition into polycarbonate membranes with nanosize pores [199,202,203,204]. Different electrodeposition techniques were studied to investigate the effect of deposition parameters on the crystallographic and magnetic properties. Similar to the majority of nanowires (e.g., Ni, Cu, Au and polypyrrole) synthesized by potentiostatic electrochemical deposition inside the PCTE membrane, Co NWs have a “cigar like” structure [191].

In the chronoamperometric method [199], the diffusion-limited current varies in time. For short times, the diffusion controlled limiting current, i, obey the Cottrell equation, i.e., i = k·t-1/2, where k is a specific constant for a given system. At longer time, radii of the diffusion zones from each nanoelectrode increase gradually, expand and overlap. In the end, the diffusion controlled limiting current is observed, when the current enters a constant steady-state. Although steady state condition is achieved during electrochemical deposition of Co, variations in deposit concentration between the pore opening and surrounding area of the recessed nanoelectrodes are observed.

**Nickel.** Polycrystalline Ni nanowires/nanotubes arrays can be designed by either electroless [205] or electrochemical deposition [189,190,191]. For electroless Ni deposition, the reducing agent in the Ni deposition was hypophosphite, which resulted in Ni-P alloy nanotubes [205] with an inner diameter and wall thickness of about 180 nm and 20 nm, respectively. These Ni NTs array have an exposed area that is over 8 times larger than a flat surface, which recommends them in sensor applications. Additionally, the nanoscale structure of these tubules leads to high redox response. For instance, the cyclic voltammetry measurements of Ni NTs electrode array have shown that the Ni(OH)_2_/NiOOH redox reaction had an electrochemical response that was 40 times larger compared to a flat surface electrode. Charge storage capacity has been found to be improved by combining overcharge oxidation of the electrode arrays with various heat treatments, which is important in advancing the performance of a nickel hydroxide electrode for nickel metal hydride batteries [205].

Nickel nanowires fabricated using the electrodeposition have been characterized for their magnetic properties. A single Ni nanowire has been grown in a single pore [179]. For current densities *i* < 10^8^ A/cm^2^, the cyclic voltammograms of a single Ni NW present a linear behavior. The electrical measurements indicated that the wire was of excellent quality, had a low contact resistance and could sustain considerably high current densities (about 3 × 10^8^ A/cm^2^). In single Ni wires, anisotropic magneto resistance was observed only when the magnetic field was applied perpendicular to wires. In this case, a change in the maximum resistance of about 1% was observed [179]. Nickel nanowires, with large aspect ratios (L/D = 1000), have around 2–3% of the typical anisotropic magneto resistance (AMR) [206]. When the array has a low-density Ni nanowire of various pore diameters enclosed in the PCTE membrane, the behavior of the resonance field vs. angle does not depend on the diameter or the density of nanowires. Moreover, the effective anisotropy field is broadened due to the presence of a substructure in the absorption spectra. The magnetization reversal of Ni nanowires was studied by anisotropic magneto resistance measurements at temperatures between 15 and 300 K [200,201] and an extra uniaxial anisotropy induced by the contraction of the membrane at low temperature has been observed.

**Copper.** Potentiostatic electrochemical template synthesis is the main method used to fabricate single crystal Cu nanowires in PCTE membranes [190,191,207,208]. The deposition process was studied in detail for different voltages [208]. The overall deposition process was found to consist of several steps that are dominated by charge transfer (at short times), transition zone (where there are compatible rates between charge transfer and diffusion), and the diffusion region. In the beginning, the length of the remaining empty pore is significantly larger than the thickness of the diffusion layers, and diffusion of ions inside the pores has a linear behavior. In time, radial diffusion of ions toward the mouth of the pore becomes significant; the diffusion layers increase, expand and overlap the neighboring pores and take over the entire surface. Overgrown NWs form caps on top of the membrane, similar to “mushrooms”. After that, linear diffusion is observed as in the case of thin films deposition.

Under controlled electrochemical conditions, single-crystalline Cu nanowires can be produced [209,210]. Additionally, single Cu nanowire has been prepared by replication of a single-ion track template [211]. A promising way of measuring nanowire properties is to use single-pore membranes. A single Cu nanowire was grown by electrochemical deposition and connected with electrodes for I–V measurements [211]. Current-voltage measurements confirmed that the Cu nanowire has an ohmic current-voltage behavior. The wire had a truncated shape and the estimated diameters were 25 and 110 nm. A low contact resistance was obtained, and the maximum current density was more than 10^8^ A/cm^2^, which also indicates the high morphological quality of nanowire.

### 4.2. Electrochemical Detection of Heavy Metals

The electrochemical treatment of wastewaters is an efficient technology as demonstrated for wastewater with high concentrations of HMIs [183]. Electrochemistry is present and assists other processes used in water treatment such as in oxidation and micro electrolysis, flotation, flocculation, coagulation. Moreover, in the case of metal hydroxides (MeO) that can adsorb pollutants by co-precipitation, MeO can be used as sacrificial anodes.

**Figure 11:**
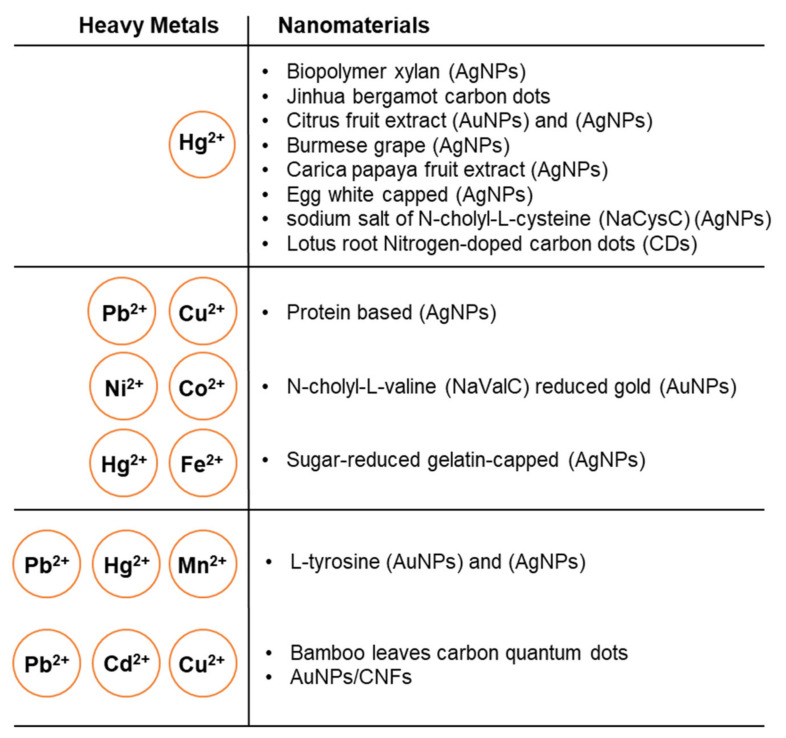
Nanomaterials used for detection of heavy metals (adapted from [212]).

Synthesis of nanostructures directly affects the mechanism and the kinetics of nanostructure growth. A better control of the process enables a better control of the morphology–structure–properties at nanoscale. This aspect becomes important in the development of low cost, large scale synthesis. Buledi et al. [212] have recently reviewed the nanomaterial-based sensors for the detection of HMIs. Figure 11 presents just an example of the versatility of nanomaterials to detect toxic heavy metals such Hg, Ni, Co, Cu Cd, Pb and Mn from waters using electrochemical methods. Figure 11 also shows that multiple heavy metals can be detected using a single nanostructured electrode. With the development of novel nanomaterials and advanced processes, the detection limits of the heavy metal ions increased owing to increased surface area and electronic conductivity.

Various methods can be employed to synthesize nanomaterials such as electrochemical deposition (In_2_O_3_ NWs [213]), electroless deposition, sol-gel (In_2_O_3_ NTs [214]), sputtering (In_2_O_3_ NRs [215]), physical evaporation [216], molecular beam epitaxy (In_2_O_3_ NWs [217]), laser ablation (In_2_O_3_ NWs [218]), in template metalorganic chemical vapor deposition (MOCVD): In_2_O_3_ NWs [219], thermal chemical vapor deposition: In_2_O_3_ [220], etc. Miniaturization of electronics and devices is the driving force in the development of integrated nanostructures 

Tubular nanostructures have intrinsic multi-functionalities that go beyond the research efforts to obtain nanotubes of various materials while posing challenges for both top-down and bottom-up approaches to nanotubes. This can be ascribed to the strong adsorption properties, large specific surface area and rich active sites of the composite. Nanotubes hold promise as candidates for highly effective nanodevices due to their four sites for attachment of other functional materials: (1) the outer surface is used for attachment, (2) the tube opening is used for loading, (3) inner surface is used for filling and (4) the interstitial region of the tube is used for doping.

Similar to multiwall CNTs, oxide nanotubes can be obtained by two different methods developed based on the template used:

- Structure-directed agents, i.e., tubular assembly of a surfactant that encapsulate oxides; similar to multiwall CNTs, oxide nanotubes obtained by this method have a multiwall structure composed of a mixture of oxide and organic components [221]. Unlike CNTs, oxide nanotubes can be obtained in gram quantities by chemistry synthesis at low temperature.
- Template directed growth: nanoporous alumina (AAO) or carbon nanotubes as templates. Polycrystalline nanotubes of ZrO_2_ [222], V_2_O_5_ [223], etc. For certain applications, the synthesis of single-crystalline oxide nanotube is required. Li et al. obtained single-crystalline In_2_O_3_ NTs [224] and single-crystalline MgO NTs [225].
- Fill in nanotubes Hollow cavities with high aspect ratio such as nanotubes can be filled in to create nanocable structures. Ajayan and Iijima were first to insert by capillarity low-melting-point metals in the hollow cavities of carbon nanotubes [226,227]. This method also works well with oxides due to their high-melting points. Li et al. [224] obtained single-crystalline In_2_O_3_ NTs loaded with metallic In by evaporating a mixture of indium and indium oxide in vacuum and single-crystalline MgO NTs filled with Ga [225]. The removal of the template without destroying the nanotubes remains an unresolved problem.

**Figure 12:**
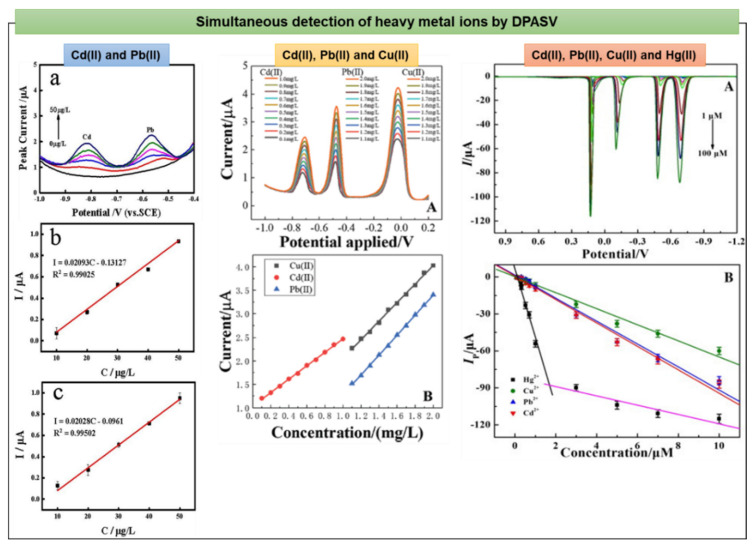
Differential pulse anodic stripping voltammetry (DPASV) curves of bismuth oxycarbide for the simultaneous detection of Cd(II) and Pb(II) (**a**-top left) [231]; thiacalix [4] arene-modified glassy carbon electrode for the simultaneous determination of tree HMIs (**b**-top middle) [232]; Hydroxyapatite-Nafion for the simultaneous detection of four HMIs (**c**-top right) [233]. The calibration curves are presented in (**b**) and (**c**) under the respective DPASV curves.

Among other methods of detection, the electrochemical methods are fast and versatile, in situ detection being also possible. The electrochemical methods for heavy metal ion detection include static techniques, potentiostatic, galvanostatic, impedance and electrochemi-luminescence. Among the potentiostatic techniques, where the applied potential is independently controlled, there are several methods that are specifically applied to the detection of heavy metal ions (HMIs). Among the potentiostatic techniques that proved very sensitive to the detection of trace HMIs is anodic stripping voltammetry (ASV) [228]. This process has a pre-concentration step, in which the HMIs are concentrated on the electrode surface. The next step is the dissolution process, in which the adatoms are oxidized back into the solution. The stripping peak current recorded during the anodic dissolution is proportional to metal concentration. The size and the quality of the electrode is very important in the detection of heavy metal ions. Figure 12 shows the differential pulse anodic stripping voltammetry (DPASV) curves obtained on bismuth oxycarbide in the simultaneous detection of 2, 3 and 4 ions. The bismuth modified electrode is considered a “green” electrode [229,230,231], has been extensively studied, and many studies have reported that adding a certain concentration of bismuth ions to the solution can improve the sensitivity of the sensor by co-deposition with heavy metal ions. The detection limits are usually lower that the national standards.

## 5. Conclusions

Water is the most important resource for living beings, and one of the greatest challenges of our time is to keep it accessible, affordable and reliable for our planet. Heavy metal ions (HMIs) are among the most dangerous pollutants. This review presents an updated overview of general chemical and physical methods with the aid of nanotechnology that are recently performed to treat and purify water based on the removal of HMIs such as As, Hg, Cd, Pb, Cr, Ni, Zn and Cu. Various electrochemical depolluting treatments are discussed such as electrodialysis, electrofiltration, electrocoagulation and recent electrochemical technologies in wasterwater treatment. Nanomaterials designed for water treatments have large specific surface area, which offers numerous and rich active sites, and strong adsorption properties. Based on the utilization of nanotechnology, there are high potential and opportunities for wastewater treatment and purification.

The interactions between toxic HMIs and nanomaterials can change the surface properties in a variety of adsorption mechanisms from electrostatic interaction to physical adsorption, to surface complexation and precipitation or ion exchange. One key area of research is the optimization of physico-chemical properties of the surface at nanoscale level so that new materials and technologies can be developed and implemented for water remediation. Wastewaters are complex systems that require several steps of treatment, for which nanomaterials can play their role and be optimized to reduce and eventually eliminate the toxic HMIs. To progress in the research on advanced materials and nanotechnologies, further studies are needed in the following directions: (I) develop inexpensive and environmentally-friendly nanomaterials and methods to functionalize their surface for unique tunable physicochemical properties, (II) develop extensive characterization methods to understand the adsorption behaviors and mechanisms of nanomaterials and to increase their effectiveness, (III) quantity and quality evaluation of the nanomaterials when used as nanoparticles-support composites, and (IV) develop customized electrochemical electrodes and techniques for complex wastewaters. Research generated by the development of technologies for HMIs removal would find direct applications in other areas of the environment field, such as air remediation and energy, due to the nature of the nanometer-scale mechanisms that these processes have in common.

## Figures and Tables

**Table 1:** Permissible limits for various heavy metals in wastewater treatment effluents according to the World Health Organization [2] and United States Environmental Protection Agency (USEPA) [1].

Heavy Metal	Permissible Limits (WHO) µg/L	Permissible Limits (USEPA) µg/L	Health Hazards
Arsenic	500	*	Carcinogenic, producing liver tumors, skin and gastrointestinal effects
Mercury	1	0.03	Corrosive to skin, eyes and muscle membrane, dermatitis, anorexia, kidney damage and severe muscle pain
Cadmium	3	10	Carcinogenic, cause lung fibrosis, dyspnea and weight loss
Lead	10	6	Suspected carcinogen, loss of appetite, anemia, muscle and joint pains, diminishing IQ, cause sterility, kidney problem and high blood pressure
Chromium	50	50	Suspected human carcinogen, producing lung tumors, allergic dermatitis
Nickel	20	200	Causes chronic bronchitis, reduced lung function, cancer of lungs and nasal sinus
Zinc	5000	*	Causes short-term illness called “metal fume fever” and restlessness
Copper	3000	*	Long term exposure causes irritation of nose, mouth, eyes, headache, stomachache, dizziness, diarrhea

* Data not available.

**Table 2:** Advantages and disadvantages of the chemical precipitation function of the coagulant used in the process.

Chemical Precipitation	Advantages	Disadvantages
Hydroxide precipitation (lime, limestone, CaCO_3_)	Easy method, simple operation, cheap and broad applications.	Accumulation of large quantity of residual sludge with water content, problematic for dewater and disposal. Not suitable for wastewaters with high heavy metals concentrations.
Sulfide precipitation	Less sludge quantity, easier dehydration.	Metallic sulfide precipitate is very small and difficult to settle down.
Ferrite co-precipitation	Efficient for heavy metals removal with density higher than 3.8 g/cm^3^ (Cu, Pb, Zn, Cd, Co, Cr, Mn, Hg, Bi, Sn, As, Mo, Fe, V, Ti). Possible separation of formed precipitates by filtration or magnetic methods.	High temperature, not suitable for large volume of wastewater, high energy consumption.

**Table 3:** The maximum adsorbents capacities for various carbon-based adsorbents.

Adsorbate	Adsorbent	Max. Adsorption, mg/g	Initial Concentration, mg/L	pH	Ref.
Pb^2+^	MWCNTs	15.6	10–80	3, 5, 7	[51]
	AC	18	10–60	5	[52]
	Oxidized MWCNTs	59		5	[52]
	SWCNTs	33.55		7	[53]
Cu^2+^	MWCNTs	12.34		7	[54]
	Oxidized MWCNTs	28.49	5–30	5	[55]
	SWCNTs	24.29		5	[52]
Cd^2+^	MWCNTs	4.1	4	3–12	[56]
	AC	2.9	4	3–12	[56]
	Oxidized MWCNTs	10.86	2–15	5	[55]
	SWCNTs	24.07		7	[53]
Ni^2+^	MWCNTs	13.05	10–80	2–9	[57]
	Oxidized MWCNTs	38.46	10–80	7	[58]
	oxidized SWCNTs	47.85	10–80	7	[58]
	GAC	26.39	10–80	7	[58]
Zn^2+^	MWCNTs	32.68	10–80		[59]
	SWCNTs	43.66	10–80		[59]
	PAC	13.04	10–80		[59]

**Table 4:** Removal efficiency of heavy metals by membrane technologies.

UF Type	MEUF					PEUF			
Heavy Metal	Cd	Ni	Zn	Pb	AsO_4_	Cd	Cu	Cr	Ni
Membrane	Polysulfone	Polycarbonate	Amicon regenerated cellulose	Ceramic	Ceramic	Polysulfone	Ceramic	Polyethersulfone	Polyethersulfone
Removal efficiency	92%	98.6%	99%	99%	19%	99%	99.5%	99.5%	100%
Reference	[80]	[81]	[82]	[83]	[83]	[84]	[85]	[86]	[87]

**Table 5:** A list of selected examples of heavy metal removal efficiencies by RO, NF and NF + RO.

Membrane	Reverse Osmosis				Nanofiltration		Reverse Osmosis + Nanofiltration
Heavy Metal	Cu	Ni	Zn	As	Cu	Cr	Cu
Removal Efficiency	99.5	99.3	98.9	As(V) 91–99%, As(III) 20–55%	96–98	99.5	95–99
Reference	[88]	[89]	[89]	[90]	[91]	[92]	[93]

**Table 6:** Removal efficiency of heavy metal ions for different anode/cathode electrode combination in electrocoagulation system.

Metals or Other Compounds	Concentration, mg/L	Electrodes Anode/Cathode	Removal Efficiency, %	References
Cr^3+^, Cr^6+^	887.2, 1495.2	Fe/Fe	100	[110]
Cu^2+^, Cr, Ni^2+^	45, 44.5, 394	Al/Fe	100	[111]
Cd^2+^	20	Al/Al	AC: 97.5 DC: 96.2	[112]
NO^3−^	150	Fe/Fe, Al/Al	90, 89.7	[113]
Pb^2+^, Zn^2+^, Cd^2+^	170, 50, 1.5	Al/SS	95, 68, 66	[114]
As	150	Al/Al, Fe/Fe	93.5, 94	[115]
TOC, Ni^2+^, Zn^2+^	173, 248, 232	SS 304-SS 304	66, 90, 100	[116]
Humic acid	20	Fe/Fe	92.69	[117]

**Table 7:** Comparison of the removal efficiency of heavy metal ions by different water treatments.

Water Treatment Method	Metal	Initial Concentration mg/L	Efficiency %	References
Reverse Osmosis (pH = 7–9)	Ni^2+^	26	99	[133]
	Cu^2+^	17	99	[133]
	Cr	167	99	[133]
Ultrafiltration (pH > 7)	Ni^2+^	50	99	[86]
	Cu^2+^	50	98	[86]
	Cr	50	93	[86]
	Ni^2+^	25	100	[134]
Nanofiltration (pH = 4–11)	Cu^2+^	200	96	[135]
Electrocoagulation (pH = 8)	Ni^2+^	394	99	[111]
	Cu^2+^	45	100	[111]
	Cr	44.5	100	[111]
	Ni^2+^, Zn^2+^	248, 270, 282, 217, 232, 236	100	[136]
Chemical Precipitation (pH > 7)	Cu^2+^, Zn^2+^, Cr^3+^, Pb^2+^	100	99.3–99.6	[13]
	Cu^2+^, Zn^2+^, Pb^2+^	0.01, 1.34, 2.3	100, >94, >92	[137]
